# Nutrient Status and Contamination Risks from Digested Pig Slurry Applied on a Vegetable Crops Field

**DOI:** 10.3390/ijerph13040406

**Published:** 2016-04-05

**Authors:** Shaohui Zhang, Yumei Hua, Liangwei Deng

**Affiliations:** 1 School of Civil Engineering and Architecture, Wuhan University of Technology, Wuhan 430070, China; 2 College of Resource and Environment, Huazhong Agricultural University, Wuhan 430070, China; ymhua@mail.hzau.edu.cn; 3 Biogas Institute of Ministry of Agriculture, Chengdu 610041, China; dengliangwei@caas.cn

**Keywords:** anaerobic digestion, land application, nutrient, heavy metal, hygienic risk

## Abstract

The effects of applied digested pig slurry on a vegetable crops field were studied. The study included a 3-year investigation on nutrient characteristics, heavy metals contamination and hygienic risks of a vegetable crops field in Wuhan, China. The results showed that, after anaerobic digestion, abundant N, P and K remained in the digested pig slurry while fecal coliforms, ascaris eggs, schistosoma eggs and hookworm eggs were highly reduced. High Cr, Zn and Cu contents in the digested pig slurry were found in spring. Digested pig slurry application to the vegetable crops field led to improved soil fertility. Plant-available P in the fertilized soils increased due to considerable increase in total P content and decrease in low-availability P fraction. The As content in the fertilized soils increased slightly but significantly (*p =* 0.003) compared with control. The Hg, Zn, Cr, Cd, Pb, and Cu contents in the fertilized soils did not exceed the maximum permissible contents for vegetable crops soils in China. However, high Zn accumulation should be of concern due to repeated applications of digested pig slurry. No fecal coliforms, ascaris eggs, schistosoma eggs or hookworm eggs were detected in the fertilized soils.

## 1. Introduction

China is one of the most important livestock breeding countries in the world. Intensive livestock production involves the release of large amounts of manure that give rise to serious environmental concerns. Approximately 3.6 billion tons of livestock manure was produced in 2007 in China [1]. Given that nowadays renewable energy development attracts extensive attention worldwide due to enormous energy demand and serious environmental pollution, anaerobic digestion processes (biogas processes) are suitable options for livestock slurry treatment due to its advantages in pollutant removal and biogas recovery. The anaerobic treatment of livestock slurries could reduce pollutants and produce biogas, but simultaneously produce lots of digested slurries [1,2,3]. These digested slurries, containing various elements and microbes, might cause secondary pollution unless they are effectively utilized or further treated.

Digested slurries are commonly used as organic fertilizers or soil amendments due to the presence of abundant organic matter (OM) and various nutrients [2,3,4]. The application of digested slurry to agricultural land cannot only enhance crop quantity and quality, but also improve the physical-chemical properties and nutrient contents of soils [5]. At the same time this could limit the heavy use of chemical fertilizers on agricultural soils. Nutrients in the digested slurries mainly consisted of N, P and K. Several studies have focused on the effect of digested slurries application on the soil OM, N and microbial community [4,6]. Plant-available P, depending on its fractions, plays an important role in crop cultivation. The water soluble P and double lactate-soluble P contents are used to assess the plant-available P in digested slurries [3]. Actually, soil P consisting of inorganic P (IP) and organic P (OP) could be further fractionated according to its availability and potential availability. Transformations among these P fractions would occur in soils fertilized with digested slurries and led to variable plant-available P [3]. However, the effects of digested slurry application on soil P availability regarding both fractions of IP and OP remain unclear.

Some potential toxic elements such as Zn, Cr, Cd, Pb, Cu and As are used extensively as feedstuff additives and are inevitably transferred to the livestock slurries [7]. Instead of decreasing during anaerobic digestion, the relative contents of heavy metals increase due to the degradation of OM in slurries. The application of digested slurries to agricultural land might consequently lead to potential heavy metal contamination [8]. It has been reported that the heavy metals contents in agricultural soils subjected to short-term application of digested slurry are usually below China’s environmental quality standards for soils [5], but the risk of heavy metals contamination deriving from long-term applications of digested slurry needs further investigation.

Besides heavy metals, environmental problems related to the application of digested slurry could also be caused by the presence of pathogens in the manure. Various pathogens and parasites are found in animal wastes. Some pathogens and parasites can still survive after these wastes are anaerobically digested and applied to land [9,10], consequently, they could potentially spread from one farm to another, contaminating crops and soils [10,11]. In order to protect the environment and humans from pathogens, it is important to understand the stability of pathogens and parasites populations, as well as the conditions after land application. Helminthes (ascaris and hookworm) infections are major concerns due to high infected populations. Schistosoma infection is another concern in the schistosomiasis epidemic prevention region. Fecal coliforms, ascaris eggs and hookworm eggs are commonly used as indicators of pathogen and parasite inactivation in biosolids and digested slurries [9]. Little information on the hygienic risk (including schistosoma infection) has been reported regarding digested slurry applications to agricultural lands in Wuhan, China.

This study involved the characteristics of digested pig slurry in regards to nutrients contents and pollutants risks. Furthermore, a 3-year investigation, involving nutrient characteristics, hygienic and heavy metals contamination risks, was conducted in a vegetable crops field in Wuhan, China.

## 2. Materials and Methods

### 2.1. Pig Slurry and Soil Sampling

Digested pig slurries and raw pig slurries, from an anaerobic digestion plant running at ambient temperature (10–30 °C), in Wuhan, China, were sampled in the four seasons of one year. This anaerobic digestion plant, with a working volume of 300 m^3^, was used to treat pig slurry with a hydraulic retention time of 8–20 days. A sub-sample of each pig slurry sample was dried at 60 °C and finely ground in a ball mill for heavy metal analysis. Soil samples, classified as yellow-brown soil, were obtained in each autumn of three sequential years. Fertilized soil samples were taken from the vegetable crops fields fertilized with diluted digested pig slurry and compound fertilizer supplement. The control soil samples were taken from adjacent vegetable crops fields fertilized only with compound fertilizer. These vegetable crops fields were under rotation cultivation of green pepper, eggplant, cabbage, cucumber, autumn lettuce and cabbage, *etc.* Each batch of soil samples, from the 0–20 cm soil layer, were obtained with sampler following a zig-zag routine. The field-moist soils were sieved (<2 mm) and stored in polyethylene bags at 4 °C. A sub-sample of each batch of soil samples was homogenized, followed by air-drying and finely ground in a ball mill for chemical analysis. All samples were collected in triplicate.

### 2.2. Nutrient and Physical Property Analysis

Organic matter, total N, total P, total K, soluble P, soluble K, NH_4_^+^-N, NO_3_^−^-N and NO_2_^−^-N contents in the slurries were analyzed according to standard analysis method [12] using non-dried slurries. Soil total porosity, electrical conductivity (EC), pH, OM, total N, total P, total K, NH_4_^+^-N, NO_3_^−^-N and available K were analyzed according to the methods described by Bao [13].

Total OP in soils was fractionated into labile organic phosphorus (LOP), moderately labile organic phosphorus (MLOP), moderately resistant organic phosphorus (MROP) and highly resistant organic phosphorus (HROP), according to Bowman-Cole method [14]. Total IP in soils was fractionated into dicalcium phosphorus (Ca_2_-P), octacalcium phosphorus (Ca_8_-P), aluminum phosphorus (Al-P), iron phosphorus (Fe-P), occluded phosphorus (O-P) and apatite (Ca_10_-P). After the air-dried and finely ground soil samples were stepwise extracted in NaHCO_3_, CH_3_COOHNH_4_, NH_4_F, NaOH-Na_2_CO_3_, Na_3_C_6_H_5_O_7_-Na_2_S_2_O_4_-NaOH and H_2_SO_4_, Ca_2_-P, Ca_8_-P, Al-P, Fe-P, O-P and Ca_10_-P fractions were determined using Mo-Sb spectrometry [15].

### 2.3. Metal Analysis

A 0.5 g dry powder of each slurry sample or soil sample was digested in HNO_3_and HClO_4_. Copper, Zn, Cr, Pb and Cd contents in the filtered supernatant were determined by graphite furnace atomic absorption spectrometry (contrAA700, Analytik Jena AG, Jena, Germany). Another 0.5 g dry powder of each slurry sample or soil sample was collected and digested in a water-bath using HNO_3_ and HCl. Arsenic and Hg in the filtered supernatant were analyzed by atomic fluorescence spectrometry (Model 8220, Beijing Jitian Co. Ltd., Beijing, China) [7].

### 2.4. Hygienic Indictor Analysis

Fecal coliforms, ascaris eggs, schistosoma eggs and hookworm eggs in the slurries and soils were counted to evaluate the hygienic risk of digested pig slurry application to the vegetable crops field. Using the non-dried slurry samples, fecal coliform enumeration in slurries were determined according to membrane filtration method. Using the non-dried soil samples, fecal coliform enumeration in soils were determined using the procedure described by Rufete *et al.* [16]. The enumeration of ascaris eggs, schistosoma eggs and hookworm eggs were examined with the saturated bitter salt solution floating method [17].

### 2.5. Statistical Methods

SPSS 18.0 software (SPSS Inc., Chicago, IL, USA) was used for statistical analysis. Paired-samples *t* test were performed to analyse the difference of the groups (*p =* 0.05).

## 3. Results and Discussion

### 3.1. Characteristics of Raw and Digested Pig Slurries

It is important to quantify pig slurry nutrition composition before field applications as the nutrients can be very variable. As shown in Table 1, the high OM, total N, total P and total K contents suggest that the digested slurry was an excellent source of organic fertilizer and could be used as a potential nutrient pool for plant growth [11]. Bedding materials such as straw and sawdust are commonly used to keep piggery warm in cold winter. Low levels of total N and total P in the raw slurry in winter probably resulted from these substrates being partly absorbed by the bedding materials. After the pig slurry was anaerobically digested, OM was partially degraded (decreased by 6%–18%) while other nutrients such as N, P and K mainly remained in the digested pig slurry. The difference between OM of the digested and raw slurries was significant (*p =* 0.041). However, no significant difference was observed for N, P and K. By comparison, lower OM, total N, total P and total K contents in the slurry were obtained in summer, which could be attributed to the dilution by more sanitary wastewater. The ratio of NH_4_^+^-N to total N in the digested pig slurry (averaged 67.4%) was higher than the raw slurry (averaged 42.9%), because part of organic nitrogen was converted to ammonia during the anaerobic digestion process. Thus the digested slurry supplies more plant-available N than raw slurry [6]. The soluble P fraction in digested pig slurry accounted for 39.2%~64.8% of total P in spring, summer and autumn, much higher than in winter (7.5%). It probably resulted from more phosphate precipitate formation due to prolonged anaerobic digestion time to guarantee treatment efficiency in cold winter [3]. It was reported that almost all the K supplied by pig slurry is highly available to crops because more than 80% of this K comes from urine in the form of water-soluble mineral salt [18]. The soluble fraction of K commonly exceeded 86% in four seasons in current study, which implies that K was an effective and available nutrition element of the digested slurry. 

Because pig waste may contain harmful pollutants, especially pathogens and heavy metals that could contribute to agricultural nonpoint source pollution, the sustainable use of pig slurry as fertilizer must start with a detailed characterization. Generally, the heavy metals contents in the digested pig slurry are higher than the raw pig slurry, because the heavy metals present in the raw pig slurry are not decreased but may be further concentrated due to mass reduction during the anaerobic digestion process [10]. No significant difference was observed between the heavy metals of digested and raw slurries in current study. The overall heavy metals (Hg, Cr, Cd, Pb, As, Zn and Cu) contents determined from the digested pig slurry, with an averaged relative contribution of Zn (69.5%) > Cu (25.9%) > Cr (3.9%) > Pb (0.58%) > As (0.12%) > Cd (0.04%) > Hg (0.001%), will inevitably cause a safety concern when applied to land. The highest Cr content during one year, assumed to derive from some feedstuff for piglet and sows [19], occurred in spring for both raw and digested slurry. The Cd, As and Hg contents in the digested pig slurry were lower than 0.16 mg·L^−1^, while Zn and Cu contents were relatively high, especially in spring and winter. Similarly, other researchers also found high contents of Zn and Cu in the digested pig slurry as these elements were added to livestock feeds on account of their antibiotic (Zn) and growth factor (Cu) properties [11]. The Zn and Cu contents in the digested pig slurry in this study were higher than those in the digested dairy slurries in other works [2], which highlights the critical role of raw slurry source on heavy metals in digested slurry. Zinc and Cu at low contents are essential micronutrients for plants, but high doses of these metals may inhibit the growth of most plant species and pose public health risks after using livestock slurries as fertilizer/amendments [2]. Thus, attention should be paid to potential Zn and Cu contamination risks when this digested pig slurry is applied to fields.

**Figure 1:**
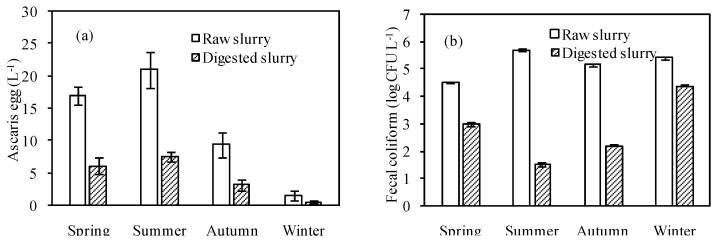
Ascaris eggs (**a**) and fecal coliforms (**b**) contents in digested and raw pig slurries in different seasons.

No schistosoma eggs were detected in both raw and digested pig slurries. Hookworm egg contents were less than 1 L^−1^ in three seasons (spring, summer and autumn) and was below the detection limit in winter in these slurries. Ascaris egg contents in spring and summer were fairly high compared to autumn and winter in both raw and digested slurries (Figure 1a). The results of the hygienic analysis obtained in current study were compared with the discharge standard of pollutants (fecal coliform ≤ 10,000 CFU·L^−1^, ascaris eggs ≤ 2 L^−1^) for livestock and poultry breeding in China (GB18596-2001). Fecal coliforms in raw pig slurry fluctuated with season (Figure 1b), but decreased drastically (by 99%) after anaerobic digestion. It met the discharge standard except in winter (25,000 CFU·L^−1^). Côté *et al.* [20] enumerated *E. coli* in 20 samples of digested pig slurries, among which *E. coli* was not detected in 15 samples and *E. coli* enumeration was reduced with 2.48–4.16 log CFU·mL^−1^ (99.67%–99.99% reduction) in the other five samples. Although the anaerobic digestion process results in an efficient removal of pathogens and parasites, ascaris eggs (or helminth eggs) remaining in digested sludge [9,21] and co-digested organic wastes [10] have been reported. Ascaris egg contents in this digested slurry exceeded discharge standards except in winter, but the anaerobic digestion process still led to decrease of ascaris egg contents by 60%–66% (Figure 1a). This result suggests anaerobic digestion reduced most fecal coliforms and parasite eggs and improved the hygienic condition [21].

### 3.2. Physical Properties and Nutrients Status of Vegetable Crops Soils Influenced by Digested Pig Slurry

As shown in Table 2, the soil total porosity percent increased from 49.4% ± 0.7% to 52.6% ± 0.8% after fertilization with digested slurries. Furthermore, the bulk density and grain size both suggested a promotion ofsoil physical properties. All three mentioned properties would combine to play a positive role on the oxygen transfer to the plants roots. Fertilized soils exhibited greater electrical conductivity (EC) than the control. The high concentration of soluble salts in slurry caused a high EC [22], which suggested that the application of pig slurry to soils may cause salinization. Applications of pig slurry decreased soil acidity to a depth of 8 cm at the rate of 20 and 40 m^3^·ha^−1^, and at the 80 m^3^·ha^−1^ rate the increase was present to a depth of 25 cm [23]. The sampling depth of soil in current study was 0–20 cm with different soil properties from previous research. The pH in the fertilized soils averaged 7.40 ± 0.06 for three years, only slightly higher than control soil, 7.27 ± 0.26.

Digested pig slurry as a fertilizer source received complimentary remarks from local farmers due to the high vegetable production and quality. Soil OM content increased significantly (*p =* 0.016), on average by 35.8% after the application of digested slurry (Table 3). Other researchers found that fertilizing with the digested cattle (or dairy) slurry did not influence the soil OM content [3,6]. This difference in soil OM content, as compared to our study, might result from different fermentation substrates, soil characteristics and crop types. Compared to control soil, total N in fertilized soil increased significantly (*p =* 0.044). Considering the large amount of total N in digested pig slurry (Table 1), the accumulation of total N in the fertilized soil was inconspicuous, and only 14% higher than control soil. This could have resulted from a loss of NH_4_^+^-N by volatilization, N uptake by crops and nitrification/denitrification [24]. Available N in soils mainly consists of NH_4_^+^-N and NO_3_^−^-N. Compared with control soil, a slight increase of NH_4_^+^-N content while a decrease of NO_3_^−^-N content (by 33.7%) in the fertilized soil were found. Decrease in NO_3_^−^-N content could be attributed to microbial assimilation of NO_3_^−^ [25] and suppression of nitrification by a high OM content in the fertilized soil. Potassium is relatively immobile in soils and is normally retained in the top soil layer when applied in soluble forms [18]. Compared with control soil, the total K contents in the fertilized soil varied slightly but the available K content increased significantly (*p =* 0.016) due to the high soluble K fraction in digested pig slurry (Table 1).

**Figure 2:**
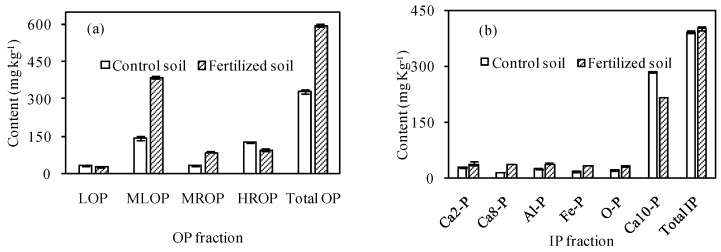
Fractionation of OP (**a**) and IP (**b**) in control and fertilized soils.

In soils, P derived from slurry is adsorbed with high binding energy at the surface of the soil mineral fraction. Adsorption occurs in the functional groups of oxides and kaolinite and mainly through the mechanism of ligand exchange [26]. However, when pig slurry is applied to soil at high rates, high total P content may increase the risks of P transference due to surface runoff. A significant increase of total P content (*p =* 0.012) was found in fertilized soil as compared to control soil (Table 3). To explore the plant-availability of the increased P content, the soil P was fractionated using soil samples fertilized with the digested pig slurry for 3 years. As shown in Figure 2, the total OP content increased (by 81.3%) while the total IP content only increased from 391.0 ± 4.2 mg·kg^−1^ to 402.3 ± 4.7 mg·kg^−1^. The LOP fraction is the smallest part of total OP and the most variable fraction over time, because it is instable and subject to changes according to management and soil-climatic conditions. The other OP fractions are more stable and resistant to alterations [27]. In this study, the LOP, MLOP, MROP and HROP contents accounted for 9.3%, 43.2%, 9.3% and 38.1% of total OP for control soil, respectively, and 4.9%, 64.5%, 14.9% and 16.0% for the fertilized soil. The OP fractions in both soils studied were in the order of MLOP > HROP > MROP > LOP. The application of digested pig slurry did not change the dominant proportions among the OP fractions. Although the LOP and HROP contents decreased respectively by 5.4% and 23.6%, substantial increase of MLOP and MROP contents (by170.5% and 182.7%, respectively) resulted in a considerable increase of total OP content. Yin *et al.* [28] reported similar results that the MLOP content in soils increased by 219.3% after the pig manure application. It was reported that the contribution of soil OP to the increase in available P (labile P) can be more relevant as the soil OM increased after organic fertilization [27]. An increase in available P (sum of LOP and MLOP) along with the increase in OM was found in the fertilized soil in current study.

Inorganic P in soils can interact with cations such as Ca, Fe, and Al, resulting in different availability and mobility. Based on plant-available IP, Ca_2_-P could be classified as the initial available P source, Al-P, Ca_8_-P and Fe-P could be classified as the second available P source, while Ca_10_-P and O-P could be classified as potential P pool because of hardly being taken up by crops. As shown in Figure 2b, the potential P pool (O-P and Ca_10_-P) contents decreased by 18.2% while those of high-availability P such as Ca_2_-P, Al-P, Ca_8_-P and Fe-P increased. Ca_2_-P could be easily taken up by crops and subsequently transformed from Ca_8_-P. A considerable increase in Ca_2_-P and Ca_8_-P contents was also found in soils fertilized with manure [29]. Increase in all available IP fractions, when soil was submitted to slurry application, may be beneficial in raising bioavailable P [30]. The low-availability P (sum of MROP, HROP, O-P and Ca_10_-P) content was 463.8 ± 2.5 mg·kg^−1^ in control soil and 434.5 ± 4.4 mg·kg^−1^ in the fertilized soil, accounting for 64.4% and 43.5% of total P, respectively. This, considerable increase in TP content but decrease in low-availability P fractions in the fertilized soil, implies that the application of digested pig slurry highly improved the plant-available P in the soils. However, caution must be taken, given that continuous applications at high rates may promote P accumulation, resulting in over-fertilization of P and eutrophication [30].

### 3.3. Heavy Metals and Hgyenic Condition of Vegetable Crops Soil Fertilized with Digested Pig Slurry

Repeated fertilization with slurry may also cause the accumulation of chemical elements. Considering most crops use only a small amount of metals to complete their life cycles, soil and water may be contaminated. As shown in Table 4, there were slight differences between the mean Hg, Cd, Pb, As and Cu contents in control and fertilized soils. The As content exceeded the maximum permissible content in control soil, and increased slightly after the application of digested pig slurry. The difference for As between the two soils types was small but statistically significant (*p =* 0.003). Therefore, more attention should be paid to the risks caused by As contamination in future work. Mercury in all the soils were typically <1 mg·kg^−1^ in the present study. Although the differences of Hg, Cd, Pb, Zn and Cu between the two soils types were not significant, the risk attributed to heavy metals accumulations should not be ignored. Copper and Zn accumulation in soils with pig slurry spreading had been widely reported, leading to phytotoxic levels that may reduce crops yields [8,31]. High Zn contents (Table 1) in digested pig slurry may have led to considerable increase in Zn content in the fertilized soil, 34.3% higher than control soil. Zinc content did not persistently increase annually and was less than the maximum permissible content for vegetable crops soils (300 mg·kg^−1^) in China’s environmental quality standard for soils (GB 15618-1995). However, repeated applications may cause a potential Zn pollution. As mentioned above (Section 3.1), high Cr content in digested slurry was found in spring, but the Cr content in the fertilized soil was significantly lower than control soil (*p =* 0.023) and below the maximum permissible content (GB 15618-1995). Future work should address the Cr content in vegetable crops and the behavior of Cr in soils. 

Bacteria present in slurry, like *E. coli* are common inhabitants of the gastro-intestinal mucosae, and are not adapted to survive in soils as their optimal growth temperature is around 37 °C [32]. As mentioned above (Section 3.1), anaerobic digestion reduced most fecal coliforms, ascaris eggs, schistosoma eggs and hookworm eggs, but the ascaris egg content still exceeded the discharge standard (GB18596-2001) except in winter. It was reported that ascaris eggs, generally used as an indicator of helminth egg inactivation, are persistent and can remain infective in the soil for years [33]. Gibbs *et al.* [34] also concluded that soils amended with biosolids could not be considered free from pathogens for at least one year following amendment. However, in current study no ascaris eggs, schistosoma eggs, hookworm eggs or fecal coliforms were detected in the soil fertilized with slurry. However, future work should address the hygienic risks of vegetable crops.

## 4. Conclusions

After anaerobic digestion, abundant N, P and K nutrients remained in the digested pig slurry, and the hygienic conditions of pig slurry were improved as well. The heavy metals contents in the pig slurries varied in the seasons, and high Cr, Zn and Cu contents in the digested pig slurry were found in spring. Digested pig slurry applied to vegetable crops field led to significant increases in OM, total N, total P and available K contents (*p* < 0.05), which suggests an improvement in soil fertility. Plant-available P in the fertilized soils increased because of a considerable increase in total P content and decrease in low-availability P fractions. The As content in the fertilized soils increased slightly but significantly (*p =* 0.003) compared with control soils. The Hg, Zn, Cr, Cd, Pb, and Cu contents in the fertilized soils did not exceed the maximum permissible contents for vegetable crops soils in China. However, repeated applications of digested pig slurry to soils may result in over-accumulation of Zn. No fecal coliforms, ascaris eggs, schistosoma eggs or hookworm eggs were detected in the fertilized soils.

## Figures and Tables

**Table 1:** Nutrients and heavy metals contents in digested and raw pig slurries in different seasons (unit: mg·L^−1^ unless specified).

Item	Raw Slurry				Digested Slurry			
	Spring	Summer	Autumn	Winter	Spring	Summer	Autumn	Winter
OM(% dry matter)	87.1 ± 10.8	42.3 ± 6.7	44.1 ± 8.0	42.9 ± 7.2	69.2 ± 8.9	24.7 ± 4.6	38.9 ± 5.9	36.2 ± 4.7
Total N	3370.6 ± 229.2	2757.9 ± 160.9	2373.3 ± 197.1	779.7 ± 157.8	2723.4 ± 109.1	1320.0 ± 145.9	1457.0 ± 124.1	1578.2 ± 181.9
Total P	904.6 ± 138.1	476.7 ± 89.7	1464.4 ± 95.8	381.9 ± 70.5	927.7 ± 93.2	167.8 ± 20.3	1067.8 ± 80.3	490.1 ± 12.9
Total K	785.3 ± 65.4	810.6 ± 90.5	891.6 ± 69.6	936.7 ± 191.7	768.1 ± 49.9	416.4 ± 38.9	698.5 ± 35.7	1284.8 ± 65.3
NH_4_^+^-N	1081.0 ± 156.8	1420.2 ± 159.3	1192.0 ± 149.6	293.9 ± 40.1	1366.6 ± 185.9	1047.5 ± 101.1	1398.7 ± 89.5	695.1 ± 90.3
NO_3_^−^-N	58.5 ± 9.9	4.5 ± 1.8	6.0 ± 1.9	28.1 ± 5.0	19.1 ± 4.8	1.1 ± 0.6	3.9 ± 0.9	6.8 ± 1.0
NO_2_^−^-N	0.1 ± 0.05	0.1 ± 0.04	0.1 ± 0.03	0.2 ± 0.04	ND	0.1 ± 0.03	0.4 ± 0.03	0.6 ± 0.06
Soluble P	399.8 ± 45.1	307.0 ± 41.0	598.7 ± 85.7	48.3 ± 14.9	363.6 ± 31.5	108.7 ± 12.9	507.2 ± 74.1	36.8 ± 6.8
Soluble K	744.3 ± 80.2	783.8 ± 115.3	850.3 ± 93.3	911.2 ± 154.5	722.5 ± 79.7	360.0 ± 27.9	681.5 ± 29.0	1109.2 ± 89.4
Hg	0.012 ± 0.04	0.016 ± 0.02	0.074 ± 0.09	0.023 ± 0.07	0.002 ± 0.01	0.001 ± 0.01	ND	0.001 ± 0.01
Cr	13.20 ± 1.23	0.01 ± 0.01	ND	ND	16.80 ± 3.89	0.10 ± 0.03	0.09 ± 0.01	ND
Cd	0.06 ± 0.01	0.02 ± 0.01	0.01 ± 0.01	0.01 ± 0.01	0.06 ± 0.02	0.02 ± 0.01	0.01 ± 0.01	0.07 ± 0.01
Pb	0.79 ± 0.11	0.62 ± 0.08	1.36 ± 0.47	0.68 ± 0.08	1.13 ± 0.55	0.23 ± 0.05	0.20 ± 0.06	0.98 ± 0.29
As	0.15 ± 0.04	0.13 ± 0.03	0.14 ± 0.02	0.11 ± 0.04	0.13 ± 0.03	0.16 ± 0.06	0.16 ± 0.03	0.09 ± 0.01
Zn	107.2 ± 15.5	20.0 ± 4.1	27.8 ± 6.6	22.0 ± 6.1	217.1 ± 52.7	7.3 ± 0.9	6.1 ± 0.7	71.6 ± 17.7
Cu	37.8 ± 7.5	9.6 ± 0.9	18.3 ± 4.3	7.2 ± 0.9	72.4 ± 15.9	10.3 ± 2.1	5.5 ± 0.9	24.2 ± 5.7

Note: ND, not detected.

**Table 2:** Physical properties of control and fertilized soils.

Item	Control Soil			Fertilized Soil		
	1a	2a	3a	1a	2a	3a
pH	7.48	6.98	7.35	7.33	7.42	7.44
EC (ms·cm^−1^)	0.831	0.619	0.725	0.915	0.844	0.896
Bulk density (g·cm^−3^)	1.33	1.36	1.32	1.31	1.29	1.30
Total porosity (%)	50.18	49.20	48.92	52.24	53.53	52.14
Grain size (%)	>5 mm: 52.0	>5 mm: 49.7	>5 mm: 52.4	>5 mm: 48.0	>5 mm: 40.8	>5 mm: 42.6
	5~2 mm: 17.6	5~2 mm: 20.4	5~2 mm: 19.4	5~2 mm: 23.6	5~2 mm: 27.5	5~2 mm: 25.7
	2~1 mm: 7.7	2~1 mm: 10.9	2~1 mm: 9.7	2~1 mm: 8.5	2~1 mm: 10.7	2~1 mm: 10.3
	1~0.5 mm: 9.4	1~0.5 mm: 9.2	1~0.5 mm: 8.5	1~0.5 mm: 9.5	1~0.5 mm: 9.6	1~0.5 mm: 10.2
	0.5~0.25 mm: 7.7	0.5~0.25 mm: 6.2	0.5~0.25 mm: 5.5	0.5~0.25 mm: 6.5	0.5~0.25 mm: 8.5	0.5~0.25 mm: 7.9
	<0.25 mm: 5.6	<0.25 mm: 3.6	<0.25 mm: 4.6	<0.25 mm: 4.0	<0.25 mm: 2.9	<0.25 mm: 3.3

**Table 3:** Nutrients contents in control soils and soils fertilized with digested pig slurry.

Item	OM (g·kg^−1^)	Total N (g·kg^−1^)	Total P (g·kg^−1^)	Total K (g·kg^−1^)	NH_4_^+^-N (mg·kg^−1^)	NO_3_^—^N (mg·kg^−1^)	Available K (mg·kg^−1^)
Control soil	15.37 ± 3.37	1.13 ± 0.06	0.78 ± 0.04	23.13 ± 1.81	4.0 ± 0.7	27.0 ± 1.8	508.7 ± 46.9
Fertilized soil	20.87 ± 2.56	1.29 ± 0.06	1.10 ± 0.10	22.80 ± 2.65	4.7 ± 1.5	17.9 ± 4.5	592.7 ± 34.9

Note: expressed in average ± SD in 3 years.

**Table 4:** Contents of heavy metals in different soils (unit: mg·kg^−1^).

Metals	Control Soil				Fertilized Soil				Maximum Permissible Content ^1^
	1a	2a	3a	Mean ± SD	1a	2a	3a	Mean ± SD	
Hg	0.42	0.17	0.31	0.30 ± 0.13	0.32	0.17	0.31	0.27 ± 0.08	1
Cr	83.6	110	91.5	95.0 ± 13.6	77.6	100	84.3	87.3 ± 11.5	250
Cd	1.7	2	1.91	1.9 ± 0.2	1.5	2.1	2.12	1.9 ± 0.4	0.6
Pb	64.5	75	69.1	69.5 ± 5.3	59.4	71	78.9	69.8 ± 9.8	350
As	38.5	30.1	34.6	34.4 ± 4.2	42.2	33.2	38.1	37.8 ± 4.5	25
Zn	139.2	189	153	160.4 ± 25.7	223.6	210	213	215.5 ± 7.1	300
Cu	92.9	90	92.7	91.9 ± 1.6	90.2	92	94.3	92.9 ± 2.1	100

^1^ Specified for vegetable crops soils in the China Environmental Quality Standard for Soils (GB 15618-1995).
